# S03 Including older adults in health enhancing physical activity: Learning lessons from implementing an evidence-based falls prevention intervention in different healthcare contexts and countries

**DOI:** 10.1093/eurpub/ckae114.206

**Published:** 2024-09-26

**Authors:** Frances Horgan

**Affiliations:** School of Physiotherapy Royal College of Surgeons in Ireland

## Abstract

The Falls Management Exercise program (FaME) is proven to reduce falls and their consequences, and increase habitual physical activity and independence up to two years post-intervention in older people at risk of falls. Many complex and interacting factors influence the availability and delivery of FaME, and its uptake and adherence by participants. Implementation studies in the UK have identified ways to increase the availability of FaME and have helped us identify its essential components for effectiveness, and the adaptable components to suit local needs. This symposium will take us through the journey from original trials, through large UK implementation studies, to the current Irish evaluation study, showing how lessons learned are being used to adapt FaME to local contexts and needs.

**Project description:**

FaME was developed and tested in the 1990's, and its implementation in the UK has been explored more recently. FaME is not widely available, and when it is, the PhISICAl Implementation Study showed it is often shortened or missing key fidelity components (eg. retraining getting up from the floor or promoting home exercise). With local commissioners, Instructors, and participants, the FLEXi implementation study identified why commissioners support FaME, its delivery and cost, and strategies to sustain FaME's fidelity and quality.

Informed by these studies, we are now establishing and evaluating FaME in Ireland. Using early sites as case-studies, we aim to optimise reach and equity, maintain effectiveness and appropriateness, and find suitable physical activity options for people after FaME.

**Conclusions:**

FaME improves physical activity for those where normally physical activity declines over time. The studies we will present will bring us from initial trials and intervention design, through to implementation of FaME fitting within local contexts and needs. It will show how FaME impacts habitual physical activity and independence while adapting to local priorities and service contexts.

